# Determination of Parabens, Bisphenol A and Its Analogs, Triclosan, and Benzophenone-3 Levels in Human Urine by Isotope-Dilution-UPLC-MS/MS Method Followed by Supported Liquid Extraction

**DOI:** 10.3390/toxics10010021

**Published:** 2022-01-06

**Authors:** Hsin-Chang Chen, Jung-Wei Chang, Yi-Chen Sun, Wan-Ting Chang, Po-Chin Huang

**Affiliations:** 1 Institute of Food Safety and Health, College of Public Health, National Taiwan University, Taipei 10055, Taiwan (R.O.C.); hsinchang@ntu.edu.tw (H.-C.C.); r06851006@ntu.edu.tw (Y.-C.S.); 2 Department of Chemistry, Tunghai University, Taichung 407224, Taiwan (R.O.C.); 3 Institute of Environmental and Occupational Health Sciences, School of Medicine, National Yang Ming Chiao Tung University, Taipei 11221, Taiwan (R.O.C.); jungwei723@gmail.com; 4 National Institute of Environmental Health Sciences, National Health Research Institutes, Miaoli 35053, Taiwan (R.O.C.); wtchang2@nhri.edu.tw; 5 Department of Medical Research, China Medical University Hospital, China Medical University, Taichung 40678, Taiwan (R.O.C.); 6 Research Center for Environmental Medicine, Kaohsiung Medical University, Kaohsiung 80708, Taiwan (R.O.C.); 7 Department of Safety, Health and Environmental Engineering, National United University, Miaoli 360302, Taiwan (R.O.C.)

**Keywords:** bisphenols, parabens, triclosan, isotope-dilution UPLC-MS/MS, biomonitoring

## Abstract

The development of a rapid analytical approach for determining levels of antibacterial agents, plasticizers, and ultraviolet filters in biosamples is crucial for individual exposure assessment. We developed an analytical method to determine the levels of four parabens—bisphenols A (BPA) and its analogs, triclosan (TCS), triclocarban, and benzophenone-3 (BP-3)—in human urine. We further measured the levels of these chemicals in children and adolescents. We used a supported liquid extraction (SLE) technique coupled with an isotope-dilution ultraperformance liquid chromatography-tandem mass spectrometry (ID-UPLC-MS/MS) method to assess the detection performance for these chemicals. Forty-one urine samples from 13 children and 28 adolescents were assessed to demonstrate the capability and feasibility of our method. An acceptable recovery (75.6–102.4%) and matrix effect (precision < 14.2%) in the three-level spiked artificial urine samples were achieved, and good performance of the validated ID-UPLC-MS/MS method regarding linearity, limits of detection, and quantitation was achieved. The within-run and between-run accuracy and precision also demonstrated the sensitivity and stability of this analytical method, applied after SLE. We concluded that the ID-UPLC-MS/MS method with SLE pretreatment is a valuable analytical method for the investigation of urinary antibacterial agents, plasticizers, and ultraviolet filters in humans, useful for human biomonitoring.

## 1. Introduction

Risks to human health have become more serious in past decades, due to increased exposure by means of the ingestion or dermal absorption of numerous old and new hazardous chemicals, such as parabens (PBs), bisphenol A (BPA) and its analogs, triclosan (TCS), and benzophenone-3 (BP-3), which are present in personal care products, consumer products, and food. To assess the exposure scenario of these ubiquitous chemicals in people, the analysis of either the substances themselves or their metabolites in noninvasive samples, such as urine, is crucial [1,2].

Methylparaben (MePB), ethylparaben (EtPB), propylparaben (PrPB), and butylparaben (BuPB) are antimicrobial preservatives derived primarily from industrial synthesis and are widely and legally used in food, pharmaceuticals, and personal care products (PCPs) because they are odorless, inexpensive to manufacture, and highly effective in preventing the growth of microbiotics (e.g., bacteria) [3,4]. In commercial products, such as PCPs, the combination of two or three PBs is usually used for preservation with the concentration of ≥0.4% for a single PB or ≤0.8% in sum for combined PBs. These PBs are classified as endocrine-disrupting chemicals (EDCs), and estrogenic activity is reportedly associated with the increased carbon number of the alkyl chain [5,6,7,8]. Several studies have also revealed their obesogenic potential [9,10] and adverse effects on animal DNA [5,11]. One epidemiologic study also indicated that PrPB is considerably associated with DNA damage in human sperm [12]. Furthermore, two antibacterial ingredients, TCS and triclocarban (TCCB), are frequently used in >2000 products, including PCPs (e.g., toothpaste and hand soap); detergents and plastics are also EDCs, and are found in a wide variety of matrixes worldwide [13,14]. Dermal absorption from PCPs is the primary route for human exposure to TCCB or TCS because of their high concentrations (0.1–0.3% [*w*/*w*]) [13,14,15]; both have been detected in human blood and urine [13,14]. Both TCS and TCCB were also associated with DNA damage and lipid peroxidation in children. This association entails that these individuals face a relatively serious health risk from these products [14,16].

BPA and its analogs (e.g., bisphenol S (BPS) and bisphenol F (BPF)) are known not only as plasticizers, but also as highly concerning EDCs; human exposure to BPA and its analogs derives primarily from food during planting, farming, production, and migration from food contact materials (FCMs), especially tin cans, which contain 20.8 ± 33.0 ng/g of BPA, a higher amount than that contained in glass or plastic materials [17,18,19]. BPA is listed in the candidate list of substances of substantial concern because of its toxicity by the European Chemical Agency (ECA), and the use of BPA in FCMs and consumer products is, therefore, limited [20]. Alternative chemicals, such as BPS and BPF, which share similar chemical structures and properties to BPA, are used more often in industry [21]. Notably, BPS is commonly used for BPA-free products, and its concentration levels in thermal receipt paper and aquatic environments are comparable to that of BPA [21,22]. Additionally, BPA, BPS, and BPF are also frequently observed in food [21,23,24,25]. The chemical BP-3, also known as oxybenzone, can naturally be present in some plants and is usually used as an ultraviolet filter with a maximum concentration of 6% in sunscreen and skincare products [26,27]. A previous study reported that BP-3, similar to PBs, exhibits obesogenic activity [28] and may cause DNA damage, one of the deleterious estrogenic responses, in human breast epithelial cells [29].

The determination of PBs, BPA and its analogs, TCS and BP-3, in human urine have been analyzed by sensitive and specific liquid chromatography-tandem mass spectrometry (LC-MS/MS) followed by various sample pretreatment approaches [30,31,32,33,34]. However, the elimination of the matrix effect during ionization, an unavoidable phenomenon, using any set of sample pretreatment protocols or chromatographic systems is complex and may cause bias in quantitative data [35,36]. The commonly used sample pretreatment approach for extracting PBs, BPA and its analogs, and TCCB in urine is solid-phase extraction (SPE) with reversed-phase sorbents [32,37,38], but the condition steps of SPE and the use of polar elution solvents, such as methanol (MeOH) and acetonitrile (ACN), could increase the possibility of eluting polar interference, which causes a matrix effect in the ionization of electrospray [39]. The application of SLE in routine sample pretreatment in LC-MS bioanalysis has increased recently [40,41]. The efficiency of SLE to remove interferences in biofluids depends on target compounds, sample matrices, and loading buffer and eluting solvents of SLE [42]. For analyzing urinary hydroxylated aromatic compounds, SLE provided good performance in eliminating interference during sample pretreatment [43]. Furthermore, the matrix effect is distinct within various lots of the same matrix, such as urine in this study, although the matrix-matched calibration curve is applied to compensate for it [35,36,44]. Among several calibration approaches applied for matrix effect compensation, the application of isotope dilution with the corresponding stable-isotope-labeled internal standards (SIL-ISTDs) of target analytes is the most effective and recommended method [36,44].

Because the presence of these six antibacterial agents, three plasticizers, and one UV-filters in the environment and human fluids is inevitable, accurately assessing the doses and risks of exposure to multiple EDCs, whether from food or the use of PCPs in our daily life, is a public concern. Thus, an isotope dilution-ultra-performance liquid chromatography-tandem mass spectrometry (ID-UPLC-MS/MS) method followed by SLE was developed and validated in this study to determine the concentrations of MePB, EtPB, PrPB, BuPB, BPA, BPS, BPF, TCS, TCCB, and BP-3 in human urine. Sample application with urine from children and adolescents was also conducted to examine the capability and feasibility of the developed ID-UPLC-MS/MS method.

## 2. Materials and Methods

### 2.1. Reagent and Chemical

Chemical standards used in this study were purchased in analytical grade, accompanied by a minimum of a certificate of analysis. The target analytes BPA and BP-3 were acquired from AccuStandard (New Haven, CT, USA); BPS, BPF, TCS, and TCCB were supplied by Toronto Research Chemicals (LGC, Manchester, NH, USA), and MePB, EtPB, PrPB, and BuPB were obtained from AlfaAesar (Thermo Fisher Scientific, Lancashire, U.K.). The SIL-ISTDs of 100 μg/mL ^13^C_12_-BPA, 1 mg/mL ^13^C_6_-MePB in MeOH, and 1 mg/mL ^13^C_6_-EtPB in MeOH were obtained from Cambridge Isotope Laboratories (Tewksbury, MA, USA); PrPB-d_7_, BuPB-d_9_, TCCB-d_4,_ TCS-d_3,_ BPS-d_8,_ and BPF-d_10_ were purchased from Toronto Research Chemicals (LGC, Manchester, NH, USA); and BP-3-d_5_ was purchased from Sigma-Aldrich (St. Louis, MO, USA). The American Chemical Society reagent grade formic acid (FA) (≥98%) was purchased from Honeywell International (Charlotte, NC, USA); 7.5 M ammonium acetate solution (NH_4_Ac_(aq)_), HPLC-grade of dichloromethane, and β-glucuronidase (≥85,000 units/mL) from *Helix pomatia* were purchased from Sigma-Aldrich (St. Louis, MO, USA); and LC-MS grade ACN and methanol were purchased from J.T. Baker (Avantor, Radnor, PA, USA). Milli-Q water (H_2_O) was produced by a Millipore Direct-Q 8 Ultrapure water system (Merck KGaA, Darmstadt, Germany). Artificial urine for method development and validation was from Fisher Scientific (Thermo Fisher Scientific, Waltham, MA, USA). For neat standards, 10,000 μg/mL of stock solution was prepared in MeOH liquid in amber glass vials. Solutions of three SIL-ISTDs in methanol from the suppliers served as stock solutions directly, and the other seven powdered SIL-ISTDs were prepared in amber glass vials with MeOH to form the concentration of 1 mg/mL. The stock solutions of native standards and their corresponding SIL-ISTDs were then diluted to the appropriate concentrations in amber glass vials with MeOH to serve as the working solutions. All stock and working solutions were stored at −20 °C in the dark.

### 2.2. Sample Collection and Preparation

A total of 41 urine samples, 13 from children (aged 7–12 years) and 28 from adolescents (aged 13–18 years), were used for evaluating the capability and feasibility of the developed method with consent from the Taiwan Environmental Survey for Toxicants (TEST) 2013 and with approval no. EC1020206 reviewed by the Research Ethics Committee of the National Health Research Institutes in Taiwan [45,46].

The sample pretreatment process of urine with isotope dilution was executed based on the previous study [47,48]. Collected urine samples stored at −80 °C were thawed at 4 °C for 24 h; 100 μL of urine sample was mixed with 20 μL of MeOH containing SIL-ISTDs, 5 μL of β-glucuronidase, and 20 μL of 1.0 M of NH_4_Ac_(aq)_ with vigorous shaking on a Vortex-2 Genie shaker (Scientific Industries, USA) for 10 s [49]. After spinning slowed, a sample was incubated at 40 °C for 1 h, and an additional 135 μL of 0.1% formic acid_(aq)_ was added to quench the hydrolysis and then mixed for extraction by the cartridge of supported liquid extraction (SLE). The extract was then eluted by 0.9 mL of dichloromethane twice and dried with an SPD-2030 SpeedVac (Thermo Fisher Scientific, Waltham, MA USA) at 30 °C and a vacuum of 5 torrs. Finally, the extract was reconstructed by adding 100 μL of MeOH and 100 μL of Milli-Q water, and it was ready for injection.

### 2.3. Analysis of UPLC-MS/MS Method

The separation of 10 target EDCs was conducted by using a Waters Acquity UPLC system with the installation of a Thermo Scientific Hypersil Gold column (50 mm × 2.1 mm, 1.9 μm) and flow rate of 0.4 mL/min. To achieve optimal ionization efficiency of the target EDCs, two sets of mobile phases were used for EDC separation and ionization. The first set of mobile phases was 0.1% of formic acid aqueous solution (A1) and ACN (B1) for four PBs, TCS, and TCCB; the second set of mobile phases was pure Milli-Q water (A2) and ACN (B1) solvent for BPA, BPS, BPF, and BP-3. The gradient elution programs for both sets of mobile phases were as follows: 80% of A1/A2 was the initial condition, held for 2 min, and decreased to 20% of A1/A2 in 2 min, to 0% of A1/A2 in 2 min, and then held for an additional 1.5 min. The gradients of A1/A2 and B1 reverted to the initial condition in 0.5 min, and then re-equilibrium of the column was achieved in 1 min; the solution was ready for another injection. Column temperature and sample tray temperature were set at 30 °C and 4 °C, respectively, and the injection volume was 10 μL.

The MS/MS acquisition in multiple reaction monitoring (MRM) mode was executed, using a SCIEX API-4000 triple quadrupole mass spectrometer equipped with an electrospray ionization (ESI) source. Except for the BP-3 with the protonated precursor ion, the other nine target EDCs were ionized in negative polarity with the precursor of [M − H]^−^. The ESI voltages for positive and negative were 4500 V and −4200 V, respectively; both positive and negative ESIs shared the same ion source temperature (450 °C), curtain gas pressure (10 psi), ion source gas 1 pressure (20 psi), and ion source gas 2 pressure (18 psi). Nitrogen was used for gases in ESI and collision gas at 4 psi in MS/MS. Table 1 lists the MS/MS parameters, including the MRM ion transitions of the precursor ion with the declustering potential (DP) and two characteristic product ions with their corresponding collision energy (CE). The dwell time for each MRM transition was 50 ms. Data acquisition and processing were performed using Analysts 1.6.2 software (SCIEX, Framingham, MA, USA).

### 2.4. Method Validation

The validation of the developed ID-UPLC-MS/MS method to examine the capability and feasibility of determining 10 target EDCs in human urine was executed based on the guidelines for the bioanalytical method validation published by the European Medicines Agency (EMA) [50]. Because so-called blank urine with the absence of target EDCs is rare, artificial blank urine was prepared as the blank matrix, with three-level spiking for method validation.

The calibration solutions, SIL-ISTDs solutions, quality control (QC) solutions were prepared as mentioned in previous studies with minor modification [47,49]. The calibrators were prepared by adding 95 μL of artificial urine and 5 μL of appropriated concentrations of target EDCs working solutions. The final 10 concentration points of three EDCs (MePB, TCS and BP-3) were 0.3, 1, 5, 10, 50, 100, 300, 750, 1100 and 1500 ng/mL in the calibrators; those for the other target EDCs were 0.3, 1, 2, 5, 10, 20, 50, 100, 300, and 500 ng/mL in the calibrators. These calibrators were treated the same as the samples to process the sample pretreatment and LC-MS/MS analysis.

The recovery and matrix effect of SLE and the linearity of the isotope dilution calibration curves were evaluated. Limits of detection (LODs), lower limits of quantification (LLOQs), and within-run and between-run accuracy and precision were evaluated to ensure the performance of the ID-UPLC-MS/MS method. Three concentration levels for validation were 3 × LLOQ for the low level, 50% of the upper limit of quantification (ULOQ) for the median level, and 75% of the ULOQ for the high level; these three levels were not at any point on the calibration curves. For linearity, a 10-level matrix-match calibration curve for each analyte was established with a range from the limit of quantification to ULOQ; the linearity was confirmed by the coefficient of determination (*R*^2^) with the weighted (1/x) linear regression calibration. LODs and LLOQs in the artificial urine were determined with a signal-to-noise ratio (S/N) of ≥3 and ≥10, respectively.

The recovery and matrix effect without the calibration of SIL-ISTDs were examined, according to relevant studies with three sets of samples: (1) neat solution standards, (2) post-spike in the artificial urine, and (3) pre-spike in the artificial urine [44]. Three concentrations—low, medium, and high levels—in three replicates (*n* = 3) for each level were applied and analyzed to assess the recovery and matrix effect. The within-run and between-run accuracy and precision were evaluated by means of the concentration values of the spike and were measured by performing five replicates of three spiked levels on the same day (*n* = 5) and three consecutive days (*n* = 5 × 3 for each level), respectively. The accuracy and precision were calculated. The validation criteria to assess the capability and feasibility of this developed method were based on EMA standards [50].

### 2.5. Quality Control

The preparation of the quality control (QC) samples was the same as that of the calibrators, except for the spiked concentrations. According to the guidance of EMA, the concentration points of the three QC levels, which should not be the concentration points of the calibrators, were 3 × LLOQ, 30–50% of ULOQ and ≥75% of ULOQ [50]. For quality control (QC), three spiked QC samples with three concentrations, used in the test of within-run and between-run assay variability, were applied at intervals of every 10 samples during sample analysis. The QC criteria of EMA were ≥85% for accuracy and ≤15% for precision [50].

## 3. Results and Discussion

### 3.1. Method Validation and Performance

The analytical characteristics of this developed ID-UPLC-MS/MS method were validated as follows, using artificial urine as the blank matrix.

#### 3.1.1. Recovery and Matrix Effect

The SLE technique employs cleaned and sized porous diatomaceous earth as the sorbent and provides acceptable recovery of target analytes with minimal interference in LC-MS bioanalysis [40,51,52]. The protocol of SLE recommended by the supplier for neutral compounds was evaluated and applied to the human urine pretreatment because of the acceptable results regarding the spiked recovery and relative standard deviation (%RSD) of target EDCs in artificial urine, with three levels utilized in the method validation and triplicates for each level [53]. The matrix effect was examined to discuss the removal of interference. The investigation of the recovery and the matrix effect was performed by examination of Abundance_set (iii)_/Abundance_set (ii)_ × 100% and (Abundance_set (ii)_/Abundance_set (i)_) × 100%, respectively, of the target native EDCs without calibration of SIL-ISTDs [44]. Table 2 lists the observed results of recovery, and the matrix effect of target EDCs in artificial urine revealed not only acceptable but also stable recoveries. The mean recoveries (%RSD) of EDC in low-, median-, and high-level concentrations were 75.6–102.4% (1.8–10.5%), 84.4–99.5% (1.9–11.1%), and 86.8–98.4% (1.7–14.1%), respectively. The recovery of most target EDCs fulfilled the criteria of EMA. The mean matrix effects for three levels of analytes spiked in artificial urine ranged from 79.5% to 118.9%, and the precisions (%RSD), measured at <14.2%, met the criteria of EMA (15% for the precision of matrix effect) [50]. Hence, the condition of SLE was applied to further validations of the ID-UPLC-MS/MS method.

#### 3.1.2. Linearity, LODs, and LOQs

Table 3 lists the results of linearity from the 10-level calibration curves of the 10 target EDCs, and the data reveal that the efficient performance of the coefficients of determination (r^2^) ≥ 0.9952 in the artificial urine met the criteria of EMA [50]. In addition, the LOD and LLOQ of each EDC evaluated with the spiked EDCs in artificial urine followed by the SLE-technique extraction were 0.1 and 0.3 ng/mL, respectively, and the ULOQs of target EDCs were selected according to their distributions in human urine. The ULOQs for MePB, TCS, and BP-3 were 1500 ng/mL, and those for the other EDCs were 500 ng/mL.

#### 3.1.3. Within-Run and Between-Run Accuracy and Precision

Regarding the within-run and between-run accuracy and precision, the within-run accuracies (precision) for low-, median-, and high-level concentrations were 91.1%–111.6% (precision ≤ 12.6%), 88.1%–112.3% (precision ≤ 11.7%), and 87.7%–112.4% (precision ≤ 13.3%), respectively; the between-run accuracies (precision) for low-, median-, and high-level concentration were 97.8%–103.4% (precision ≤ 6.3%), 95.5%–104.4% (precision ≤ 9.9%), and 97.5–105.8% (precision ≤ 7.8%), respectively (Table 4). The results of the within-run and between-run accuracy and precision satisfied the criteria of EMA, and the validated ID-UPLC-MS/MS method applied prior to SLE pretreatment was then applied to determine the target EDCs in human urine.

### 3.2. Application to Human Urine

A total of 41 urine samples, 13 from children and 28 from adolescents, were used to examine the capability and feasibility of this ID-UPLC-MS/MS method. Because the matrix effects varied among urine samples from each participant, the coefficient of variation (CV) of the abundance of SIL-ISTD was examined, and CVs of 10 isotope-labeled EDCs in the urine of children and adolescents were 8.8–12.4% and 9.6–11.6%, respectively, which indicated that the quantitation results would be more confident with the isotope dilution method, even with consideration of the acceptable variation among samples.

**Figure 1:**
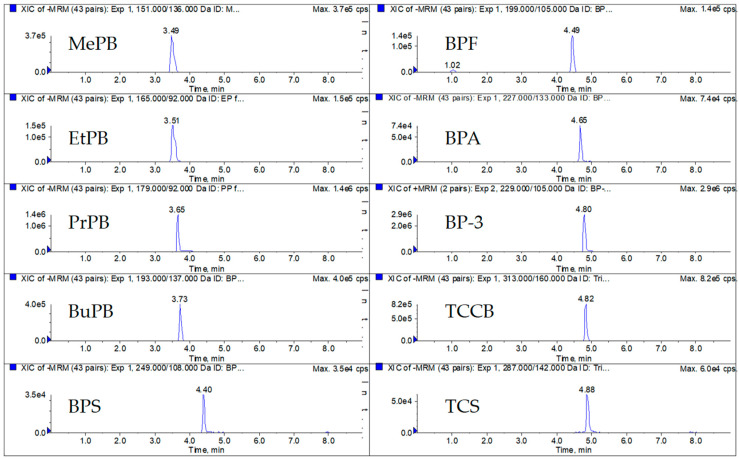
UPLC-MS/MS method chromatograms of target EDCs in an adolescent urine sample.

Figure 1 depicts the UPLC-MS/MS method chromatograms of 10 EDCs in one specimen of human urine. Regarding the distribution of the 10 EDCs in the collected urine, the preliminary results indicated that MePB, EtPB, and PrPB were the primary EDCs among the targets for the samples of both children and adolescents, and the distributions of 10 EDCs in both participant groups were similar (Table 5). The mean values of MePB, EtPB, and PrPB in the samples of the children group were 481.0 ± 246.5, 181.9 ± 241.2, and 121.3 ± 69.4 ng/mL, respectively; those of MePB, EtPB, and PrPB in the samples of the adolescent group were 435.4 ± 244.3, 177.8 ± 251.5, and 107.8 ± 73.1 ng/mL, respectively. The high levels of these three EDCs in the samples of children and adolescents might be a result of both the use of PCPs [54] and the intake of certain types of food, such as sauces [55]. In addition, the mean concentrations of TCS, TCCB, and BP-3 were 31.4, 10.5 and 9.6 ng/mL, respectively, in the children group; those were 25.4, 8.7, and 9.1 ng/mL, respectively, in the adolescent group. For BPA and its analogs, BPF and BPS, the concentration levels of BPA and BPF were similar to the mean values of 4.6 and 8.5 ng/mL, respectively, in the children group, and 3.9 and 7.0 ng/mL, respectively, in the adolescent group. The concentrations of BPS in children and adolescents were 2.0 ± 0.9 and 1.9 ± 1.3 ng/mL, respectively. The distribution of BPA in the children group was slightly lower than that in a Taiwanese study conducted by Chang et al. [56].

## 4. Conclusions

This study formulated a sensitive and stable ID-UPLC-MS/MS method applied prior to SLE for determining MePB, EtPB, PrPB, BuPB, BPA, BPF, BPS, TCS, TCCB, and BP-3 levels in human urine. The SLE technique used in this study also revealed (1) its efficiency in removing interference by use of a nonpolar extraction solvent and (2) the simplicity of the extraction process. The results of the validation of this method regarding recovery, matrix effect, linearity, LOD, LOQ, and within-run and between-run precision and accuracy also indicated its promise in human biomonitoring. The isotope dilution approach could improve the accuracy of quantitation results to calibrate the variation of matrixes among urine samples from each participant. This method could also be applied for further investigation of exposure scenarios to EDCs and their risk to humans in daily life.

## Figures and Tables

**Table 1:** MS/MS parameters for parabens, bisphenols, triclosan and benzophenone and corresponding SIL-ISTDs ^3^.

Analyte	MRM Transition Ions (*m*/*z*)				MRM Transition Ions (*m/z*)	
	Precursor Ion (DP ^1^, V)	Quantitated Ion (CE ^2^, V)	Qualified Ion (CE, V)	SIL-ISTD ^3^	Precursor Ion	Quantitated Ion (CE, V)
MePB	[M − H]^−^ 151 (60)	92 (27)	136 (21)	^13^C_6_-MePB	[M − H]^−^ 157	98 (28)
EtPB	[M − H]^−^ 165 (40)	92 (29)	136 (22)	^13^C_6_-EtPB	[M − H]^−^ 171	98 (29)
PrPB	[M − H]^−^ 179 (60)	92 (28)	136 (22)	PrPB-d_7_	[M − H]^−^ 186	92 (28)
BuPB	[M − H]^−^ 193 (60)	92 (32)	136 (24)	BuPB-d_9_	[M − H]^−^ 202	92 (32)
TCS	[M − H]^−^ 287 (55)	35 (30)	142 (27)	TCS-d_3_	[M − H]^−^ 290	35 (30)
TCCB	[M − H]^−^ 313 (42)	126 (28)	160 (36)	TCCB-d_4_	[M − H]^−^ 317	130 (28)
BPA	[M − H]^−^ 227 (40)	212 (25)	133 (45)	^13^C_12_-BPA	[M − H]^−^ 239	223 (25)
BPS	[M − H]^−^ 249 (70)	108 (27)	92 (40)	BPS-d_8_	[M − H]^−^ 257	112 (28)
BPF	[M − H]^−^ 199 (85)	93 (26)	105 (35)	BPF-d_10_	[M − H]^−^ 209	97 (26)
BP-3	[M + H]^+^ 229 (71)	105 (36)	71 (36)	BP-3-d_5_	[M + H]^+^ 234	110 (35)

^1^ DP = declustering potential; ^2^ CE = collision energy in MS/MS; ^3^ SIL-ISTDs: stable-isotope-labeled internal standards.

**Table 2:** Recovery and matrix effect of target EDC spike in artificial urine.

Analyte	Spike Conc. (ng/mL)	Recovery (*n* = 3)		Matrix Effect (*n* = 3)	
		Mean	RSD	Mean	RSD
MePB	0.9	100.9%	6.1%	92.4%	8.2%
	700	84.4%	1.9%	96.7%	6.1%
	1125	95.2%	1.7%	89.5%	8.6%
EtPB	0.9	91.6%	6.7%	85.6%	4.7%
	250	99.5%	7.1%	80.3%	4.8%
	375	90.8%	12.8%	88.7%	6.2%
PrPB	0.9	97.2%	5.4%	83.0%	3.8%
	250	91.8%	2.2%	92.6%	7.0%
	375	86.8%	13.7%	90.5%	11.2%
BuPB	0.9	99.3%	10.5%	85.4%	14.2%
	250	86.0%	6.9%	91.8%	13.2%
	375	98.4%	13.3%	88.4%	13.9%
BPA	0.9	85.0%	3.8%	95.3%	5.1%
	250	89.1%	4.3%	79.5%	5.5%
	375	93.7%	6.2%	81.4%	5.0%
BPS	0.9	81.0%	4.6%	99.0%	6.4%
	250	89.4%	11.1%	87.7%	0.6%
	375	90.5%	5.6%	87.3%	0.3%
BPF	0.9	75.6%	1.8%	103.2%	4.0%
	250	93.0%	8.6%	81.3%	3.7%
	375	90.0%	8.4%	86.3%	4.7%
TCS	0.9	98.5%	8.9%	110.2%	2.2%
	700	89.1%	4.9%	114.7%	1.8%
	1125	92.6%	14.1%	102.2%	7.6%
TCCB	0.9	102.4%	5.8%	115.0%	7.7%
	250	92.6%	2.8%	115.8%	5.3%
	375	94.3%	9.2%	106.2%	12.0%
BP-3	0.9	90.9%	9.2%	118.9%	7.2%
	700	96.9%	8.4%	100.7%	3.9%
	1125	92.2%	11.8%	111.9%	9.1%

**Table 3:** Linearity, LOD, LLOQ, and ULOQ in artificial urine.

Analyte	*r* ^2^	Equation (1/x Weighting)	LOD (ng/mL)	LLOQ (ng/mL)	ULOQ (ng/mL)
MePB	0.9978	y = 0.2182x − 0.0670	0.1	0.3	1500
EtPB	0.9955	y = 0.1986x − 0.0537	0.1	0.3	500
PrPB	0.9982	y = 0.1625x − 0.0451	0.1	0.3	500
BuPB	0.9972	y = 0.1869x − 0.0541	0.1	0.3	500
TCS	0.9958	y = 0.1362x − 0.0284	0.1	0.3	1500
TCCB	0.9983	y = 0.1761x − 0.0179	0.1	0.3	500
BPA	0.9985	y = 0.0954x − 0.0275	0.1	0.3	500
BPS	0.9981	y = 0.0824x − 0.0228	0.1	0.3	500
BPF	0.9952	y = 0.0964x − 0.0236	0.1	0.3	500
BP-3	0.9976	y = 0.1462x − 0.0378	0.1	0.3	1500

**Table 4:** Within-run and between-run accuracy and precision.

Analyte	Spiked Conc. (ng/mL)	Within-Run (*n* = 5)			Between-Run (*n* = 5 × 3)		
		Mean_Measured_ ± SD (ng/mL)	Accuracy (%)	Precision (%)	Mean_Measured_ ± SD (ng/mL)	Accuracy (%)	Precision (%)
MePB	0.9	1.0 ± 0.1	111.6	10.4	0.9 ± 0.04	103.4	3.9
	700	754.9 ± 76.2	107.8	10.1	704.7 ± 15.9	100.7	2.3
	1125	986.9 ± 131.4	87.7	13.3	1152.9 ± 89.6	102.5	7.8
EtPB	0.9	0.9 ± 0.1	100.9	12.6	0.9 ± 0.1	97.8	6.3
	250	225.0 ± 18.8	90.0	8.3	245.2 ± 18.1	98.1	7.4
	375	386.6 ± 15.9	103.1	4.1	395.0 ± 18.4	105.3	4.7
PrPB	0.9	1.0 ± 0.1	110.5	7.7	0.9 ± 0.1	100.8	6.3
	250	236.7 ± 2.8	94.7	1.2	238.8 ± 3.3	95.5	1.4
	375	332.7 ± 14.7	88.7	4.4	370.7 ± 11.9	98.9	3.2
BuPB	0.9	0.9 ± 0.1	100.8	7.0	0.9 ± 0.04	101.7	4.1
	250	280.7 ± 12.1	112.3	4.3	252.6 ± 9.0	101.0	3.6
	375	379.5 ± 8.0	101.2	2.1	365.7 ± 14.6	97.5	4.0
BPA	0.9	1.0 ± 0.03	105.6	2.7	0.9 ± 0.02	98.3	2.8
	250	220.3 ± 11.1	88.1	5.0	259.8 ± 19.4	103.9	7.5
	375	421.7 ± 37.1	112.4	8.8	383.8 ± 16.2	102.4	4.2
BPS	0.9	1.0 ± 0.01	110.1	1.1	0.9 ± 0.02	100.4	1.9
	250	275.9 ± 30.6	110.4	11.1	261.1 ± 12.7	104.4	4.9
	375	415.0 ± 18.7	110.7	4.5	396.6 ± 16.3	105.8	4.1
BPF	0.9	1.0 ± 0.04	109.1	3.8	0.9 ± 0.03	101.0	3.0
	250	267.8 ± 31.3	107.1	11.7	245.5 ± 12.9	98.2	5.3
	375	365.4 ± 18.7	97.4	1.6	376.9 ± 15.9	100.5	4.2
TCS	0.9	0.9 ± 0.1	99.8	5.6	0.9 ± 0.01	100.7	0.8
	700	729.6 ± 30.3	104.2	4.2	675.3 ± 47.1	96.5	7.0
	1125	1095.3 ± 18.7	97.4	9.1	1138.0 ± 62.9	101.2	5.5
TCCB	0.9	1.0 ± 0.04	106.4	3.8	0.9 ± 0.01	100.7	0.6
	250	261.3 ± 15.2	104.5	5.8	243.4 ± 11.9	97.4	4.9
	375	416.3 ± 29.6	111.0	7.1	374.5 ± 14.7	99.9	3.9
BP-3	0.9	0.8 ± 0.03	91.1	3.8	0.9 ± 0.02	98.3	2.5
	700	714.1 ± 44.2	102.0	6.2	688.8 ± 64.4	98.4	9.9
	1125	1031.3 ± 57.7	91.7	5.6	1141.7 ± 73.1	101.5	6.4

**Table 5:** Distribution of parabens, bisphenols, triclosan and benzophenone in urine of children and adolescence.

Subjects		Concentration (ng/mL)									
		MePB	EtPB	PrPB	BuPB	BPA	BPF	BPS	TCS	TCCB	BP-3
Child (*n* = 13, 8–12 years old)	Min.	209.2	10.6	39.2	3.2	1.2	3.9	0.8	7.4	3.4	5.4
	Max.	925.3	703.7	281.9	13.7	10.1	16.4	3.5	86.1	22.5	16.4
	Mean	481.0	181.9	121.3	7.0	4.6	8.5	2.0	31.4	10.5	9.6
	SD	246.5	241.2	69.4	3.0	2.8	3.6	0.9	23.9	6.4	3.4
Adolescent (*n* = 28, 13–18 years old)	Min.	78.1	0.3	17.4	0.7	0.2	1.1	0.5	0.1	1.7	1.8
	Max.	959.4	1200.9	343.7	9.4	13.1	20.3	5.9	102.4	26.1	24.1
	Mean	435.4	177.8	107.8	5.2	3.9	7.0	1.9	25.4	8.7	9.1
	SD	244.3	251.5	73.1	3.1	3.7	4.4	1.3	25.4	6.2	5.4

## Data Availability

The data presented in this study are available on request from the corresponding author.
